# Visible label-free detection of bacterial DNA using flocculation of sterically stabilised cationic latexes†

**DOI:** 10.1039/d2tb02714c

**Published:** 2023-03-14

**Authors:** Elisabeth Trinh, Kate L. Thompson, Shang-Pin Wen, Gavin J. Humphreys, Bianca L. Price, Lee A. Fielding

**Affiliations:** a Department of Materials, School of Natural Sciences, The University of Manchester Oxford Road Manchester M13 9PL UK lee.fielding@mancheter.ac.uk; b Division of Pharmacy and Optometry, School of Health Sciences, Faculty of Biology Medicine and Health, The University of Manchester Oxford Road Manchester M13 9PT UK; c Division of Pharmacy and Optometry, Lydia Becker Institution of Immunology and Inflammation, Faculty of Biology Medicine and Health, The University of Manchester Oxford Road Manchester M13 9PT UK; d Henry Royce Institute, The University of Manchester Oxford Road Manchester M13 9PL UK

## Abstract

The current gold standard diagnostic for bacterial infections is the use of culture, which can be time consuming and can take up to five days for results to be reported. There is therefore an unmet clinical need for a rapid and label free alternative. This paper demonstrates a method of detecting the presence of amplified DNA from bacterial samples using a sterically-stabilised, cationic polymer latex and widely available equipment, providing an accessible alternative DNA detection technique. If DNA is present in a sample, successful amplification by polymerase chain-reaction (PCR) results in the amplified DNA inducing flocculation of the polymer latex followed by rapid sedimentation. This results in a visible and obvious change from a milky-white dispersion to a precipitated latex with a colourless and transparent supernatant, thus giving a clear visual indication of the presence or absence of amplified DNA. Specifically, the response of four polymer latexes with different morphologies to the addition of amplified bacterial DNA was investigated. Cationic latexes flocculated rapidly whereas non-ionic and anionic latexes did not, as judged by eye, disc centrifuge photosedimentometry (DCP), and UV-visible spectrophotometry. The stability of several cationic latexes with different morphologies in typical PCR reagents was investigated. It was found that unwanted flocculation occurred for a latex with a non-ionic core and a cationic corona (poly[2-vinyl pyridine-*b*-benzyl methacrylate], prepared by polymerisation-induced self-assembly) whereas a ∼700 nm PEGMA-stabilised P2VP latex (non-ionic stabiliser, cationic core), prepared by emulsion polymerisation remained stable. The sensitivity and rate of sedimentation of the PEGMA-stabilised P2VP latex was demonstrated by varying the sequence length and concentration of amplified DNA from *Pseudomonas aeruginosa* using universal bacterial primers. DNA concentrations as low as 0.78 ng μl^−1^ could readily be detected within 30 minutes from the addition of amplified DNA to the latex. Furthermore, the specificity of this method was demonstrated by showing a negative result occurs (no flocculation of the latex) when PCR product from a fungal (*Candida albicans*) sample using bacterial primers was added to the latex.

## Abstract 2

This paper demonstrates a method for detecting the presence of amplified DNA from bacterial samples using a sterically-stabilised, cationic polymer latex and widely available equipment, providing an accessible alternative DNA detection technique.

## Graphical abstract

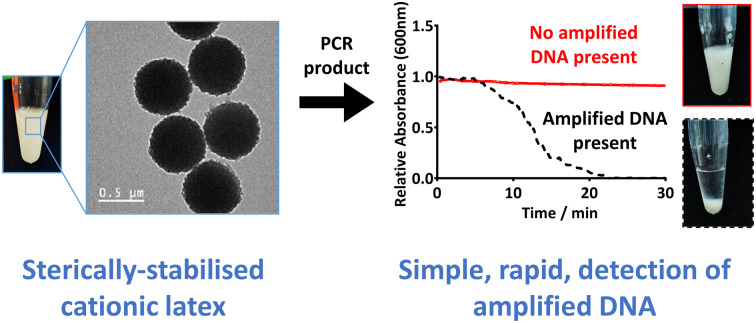

## Introduction

There remains an unmet need for a rapid diagnostic device for quick detection and identification of bacteria and other pathogens.^1–4^ Infectious diseases are often illnesses which can spread quickly and possibly lead to epidemics.^5^ It is critical to quickly diagnose initial infections and prevent further spread by diagnosing these infections *in vitro*. However, current detection strategies such as culture and serology have exhibited a lack of balance with regards to their accuracy, time consumption, and portability.^2,6–9^ Delayed identification of the causative organism and culture susceptibilities may often be responsible for delays in optimal antimicrobial therapy.^2,3^ The current gold standard technique for bacterial diagnosis is culture which can take an extended period of time of up to 72 hours.^9,10^ For sterility testing, current techniques can take up to 14 days.^11^ Therefore, there is an unmet need for a simple to use, point of contact test to determine the nature of the infection in the short term to improve the immediate diagnosis and treatment of an infection.^12–14^

There are currently various proposed and applied ways of detecting bacteria as an alternative to culture. A number of techniques for detecting the presence of specific bacterial DNA use amplification techniques to recognise and amplify the target DNA sequence using specific primers. These methods include loop-mediated isothermal amplification (LAMP) and polymerase chain reaction (PCR), the latter of which is currently the most used and researched method for amplification of nucleic acids.^15–18^ A Nucleic Acid Amplification Test (NAAT) such as PCR involves the extraction, amplification and consequent detection of DNA using a variety of methods in order to determine the presence or absence of an infection.

PCR takes approximately 2–4 hours and a very low concentration of DNA can be amplified to detectable levels, typically nanogram quantities.^19,20^ However, PCR can be time consuming, particularly compared to LAMP, and requires specialised equipment and expertise.^21,22^ This can limit the time to diagnosis meaning patients are often empirically treated with antibiotics when not needed, or alternatively are lost to follow up (*i.e.* when positive results are available but patients do not return for treatment).^13^

After the nucleic acids present in a sample have undergone amplification by conventional PCR, samples are then typically analysed using gel electrophoresis. This is the most commonly used method in research and routine laboratories for detection of DNA.^23,24^ Gel electrophoresis is known to be an efficient and effective way of separating nucleic acids and detecting the presence of DNA fragments. However, it is relatively labour intensive, requires additional equipment and can take over an hour for separation and analysis.^23,25^ Alternatively qPCR^26,27^ directly analyses amplified DNA using fluorescent labelling (such as SYBR green) but requires a more complex PCR set-up.^23,25^ There is therefore an interest in developing alternative methods of detecting amplified DNA using biosensors, which is the focus herein.

Biosensors can be involved in specific areas of the DNA detection process by improving the yield and purity of the extraction prior to the DNA reaction, or act as a DNA detection method for the PCR product.^28–30^ Their use can simplify and improve the experimental steps and/or improve the sensitivity and specificity of detecting DNA.^28^ A number of biosensors have been developed which allow ‘label free’ detection of DNA. Many of these involve the binding of DNA oligonucleotides to nanoparticles. Typically, this involves using gold or silver nanoparticles where complementary binding causes a shift in the surface plasmon resonance of the nanoparticles, resulting in a subtle but detectable (*e.g.*, *via* eye or plate reader) colour change.^31,32^ Magnetic particles have also been used in order to detect the presence of DNA *via* aggregation.^28^ In this case, amplified DNA was added to magnetic beads which bound to their surface. When placed in a magnetic field, aggregation was induced, providing label-free detection of DNA. Aggregation *via* bridging flocculation of magnetic nanoparticles has also been described by Wee *et al*.^33^ to detect pathogenic DNA. In this case, a low pH buffer was used to trigger flocculation of these magnetic particles after the addition of amplified DNA. All the aforementioned techniques speed up the time taken in order to show a positive PCR result when compared to gel electrophoresis and have the benefit of an easy-to-read visual change. However, they typically use multiple step processes including washing steps which can add to the length of the process, magnetic fields which adds complexity, or require the use of sequence specific DNA oligonucleotides. Hence, it is important to develop a quick, sensitive and easy to read approach to detect amplified DNA after PCR which does not rely on complex additional equipment.

**Scheme 1:**
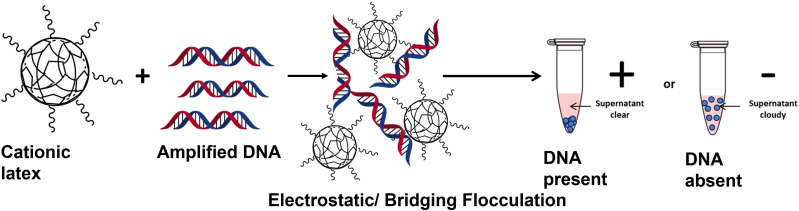
Detection of amplified DNA *via* electrostatically induced bridging flocculation of cationic polymer latex. The addition of amplified DNA to a sterically-stabilised P2VP latex causes flocculation and subsequent sedimentation of the milky white latex providing a rapid and visible method for detecting the success of a PCR. When a PCR is unsuccessful no amplified DNA will be present and no sedimentation will be observed.

This report investigates the detection of amplified DNA using a readily synthesised and scalable polymer latex. The amplification of bacterial DNA was achieved through conventional PCR using a common bacteria strain (*Pseudomonas aeruginosa*) and universal primers, which target the 16s rDNA gene of most bacterial species.^34^ DNA was amplified and then added to a series of latexes with varying morphology and functionality. For sterically stabilised, lightly cross-linked, poly(2-vinylpyridine)-based latexes, the anionic amplified DNA undergoes charge complexation with the cationic latex and causes bridging flocculation of the particles (Scheme 1). The subsequent sedimentation of the particles gives an obvious visible change which could be interpreted by a non-professional as a clear binary result indicating the presence of any amplified bacterial DNA. This has been investigated in terms of specificity, sensitivity and timescale of sedimentation using a combination of UV-visible spectrophotometry (UV-Vis) and disc centrifuge photosedimentometry (DCP).

## Results and discussion

### Addition of (co)polymer latexes to amplified DNA

The structure of DNA is often described as a double helix, with nitrogenous bases at the centre of the double helix, and a 3′-5′ phosphate backbone on the outside of the helix, which joins the individual nucleotides.^35^ Importantly, these distinctive 5′ and 3′ ends have a different structure, consisting of a hydroxyl group at the 3′ ends (3′OH) and a phosphate group at the 5′ end (5′PO_4_). These phosphate groups confer one negative charge per residue giving DNA a net anionic charge.^36,37^

A series of polymer latexes was therefore designed to investigate their association with amplified DNA *via* electrostatic interactions and determine whether they sediment over a short timescale as a result of bridging flocculation. Two different cationic latex morphologies were synthesised, along with a non-ionic and an anionic control (see Table 1). Specifically, poly(2-vinyl pyridine-*b*-benzyl methacrylate) (herein denoted ‘P2VP_32_-*b*-PBzMA_300_’) particles were prepared *via* RAFT-mediated polymerisation-induced self-assembly (PISA) to provide particles with a cationic corona and hydrophobic latex core. In contrast, lightly cross-linked PEGMA-stabilised (average *M*_*n*_ 2000 g mol^−1^) poly(2-vinyl pyridine) (herein simply referred to as ‘PEGMA-P2VP’) latexes were prepared by conventional emulsion polymerisation to yield latexes with a hydrophilic (but non-ionic) steric stabiliser and cationic core. Anionic poly(4-styrene sulfonate-*b*-benzyl methacrylate) (denoted as ‘PSS_54_-*b*-PBzMA_100_’) and non-ionic poly(benzyl methacrylate) (denoted as ‘PBzMA_200_’) were prepared by RAFT-aqueous emulsion polymerisation and RAFT miniemulsion polymerisation using a non-ionic surfactant, respectively (see ESI† for detailed descriptions).

**Table 1:** Summary of composition, size and net charge for polymer latexes investigated

Latex composition^a^	Intensity-average diameter (nm)^b^	Polydispersity index^b^	Weight- average diameter (nm)^c^	Charge^d^
PBzMA_200_	330	0.12	379	Non-ionic
PSS_54_-*b*-PBzMA_100_	660	0.13	837	Anionic
P2VP_32_-*b*-PBzMA_300_	166	0.02	242	Cationic corona
PEGMA-stabilised P2VP	754	0.06	710	Cationic core

a Latex preparation details and characterisation data can be found in the ESI (Fig. S1 to S4).

b Determined *via* dynamic light scattering (DLS) at 25 °C. All measurements were performed in triplicate on 0.01% w/w dispersions for DLS.

c Measured by disc centrifuge photosedimentometry (DCP) at 20 °C.

d Based on aqueous electrophoresis measurements and synthetic method.

**Fig. 1:**
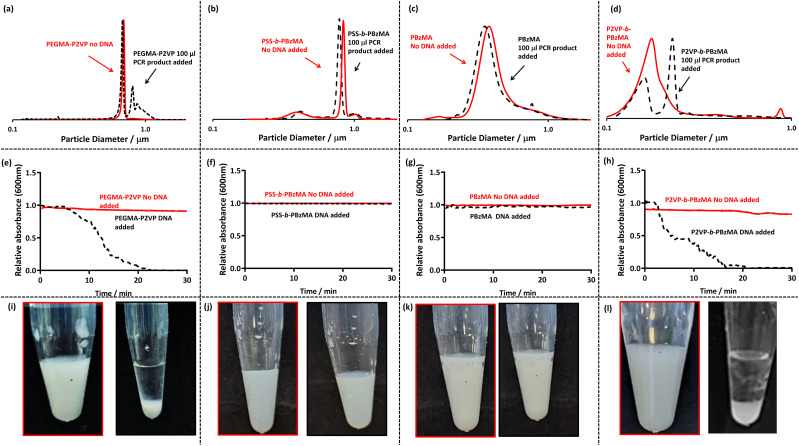
(a–d) DCP particle size distributions obtained for four (co)polymer latexes in the absence and presence of amplified PCR product at an overall DNA concentration of 3.1 ng μl^−1^ and latex concentration of 0.01% w/w. (e–h) UV-Vis spectrophotometry absorbance at 600 nm as a function of time for latexes (red) and after the addition of DNA to the latex (black). Latex particles were at a concentration of 0.1% w/w and 50 μl of purified amplified PCR product was added to observe flocculation. (i–l) Digital images of the latexes before (left) and 30 min after the addition of amplified DNA (right) at latex concentrations of 0.2% w/w and a DNA concentration of 3 ng μl^−1^.

In order to investigate if DNA induced flocculation of the four latex morphologies, amplified DNA was prepared by conventional colony PCR from a PA01 reference strain of *Pseudomonas aeruginosa* targeting the bacterial 16s gene using universal primers (27F 1492R), which amplify the DNA of most bacterial species (Table 2). This amplified PCR product is approximately 1400 base pairs in length. The amplified DNA was added to diluted latex to give an overall concentration of DNA between 2–4 ng μl^−1^ and a latex particle concentration of 0.2% w/w. This mixture was left undisturbed for 30 minutes to observe sedimentation by visual observation (Fig. 1, bottom row). Additionally, complementary experiments were performed using DCP, to evaluate the degree of incipient flocculation by analysing the observed particle size distribution (Fig. 1, top row), and UV-Vis to monitor the rate of sedimentation (Fig. 1, middle row).

**Table 2:** Details of ‘Universal’ bacterial primers used to amplify DNA *via* PCR

Primer	Target gene	Amplicon length/base pairs	Selectivity
27F, 1492R	16s rDNA	∼1400	Most bacterial species
8FPL1, 806R	16s rDNA	∼800	Most bacterial species
HDA1, HDA2	V2-V3 region of 16s rDNA	∼200	Most bacterial species

When observing the bottom row of Fig. 1, it is apparent that sedimentation occurs for both P2VP_32_-*b*-PBzMA_300_ (cationic corona, Fig. 1l) and PEGMA-P2VP (cationic core, Fig. 1i) leaving a transparent and colourless supernatant and milky white sediment. However, for PSS_54_-*b*-PBzMA_100_ (anionic corona) and PBzMA_200_ (non-ionic particles) no visible change is observed on the addition of amplified DNA and the dispersions remain milky-white (Fig. 1j and k). When investigated further using DCP (Fig. 1a–d) it is apparent that all of the initial particle size distributions (red traces) are unimodal and relatively narrow. However, on the addition of DNA, shown as black dotted lines in Fig. 1a to d, there are effectively no changes to the traces for PSS_54_-*b*-PBzMA_100_ and PBzMA_200_, indicating no incipient flocculation occurs on the addition of amplified DNA to these anionic and non-ionic particles. Furthermore, when monitoring these samples using UV-Vis by mixing the particles and DNA within a cuvette and immediately monitoring the absorbance at 600 nm for 30 minutes (Fig. 1f and g), there is no sign of sedimentation (which would be indicated by a decrease in absorbance). Thus, it can be concluded that these anionic and non-ionic particles are not suitable for detecting amplified DNA using this methodology. This was expected as DNA is anionic, meaning that electrostatically induced flocculation would not occur for anionic and/or non-ionic particles.

The black dotted DCP traces in Fig. 1a and 1d show the particle size distributions on the addition of amplified DNA to PEGMA-P2VP and P2VP_32_-*b*-PBzMA_300_, respectively. In both cases the particle size distributions show clear signs of flocculation through the appearance of peaks at larger particle sizes. This is expected as the relatively high molecular weight and negatively charged DNA is capable of electrostatically associating with the cationic latexes and causing charge neutralisation as well as bridging flocculation. This observation provides a colloidal length scale explanation of the macro-scale visual observations made in the bottom row of Fig. 1. Furthermore, when amplified DNA was added to these latexes and monitored by UV-Vis, a gradual decrease in the absorbance is observed over the 30 minute timescale (Fig. 1e and 1h). Once the particles have sedimented at the bottom of the cuvette this leaves a relatively transparent supernatant and thus a comparatively low absorption at 600 nm. These measurements also demonstrate that a 30 minute timescale is appropriate for subsequent experiments to judge whether DNA amplification has been successful or not.

### Addition of cationic latexes to individual PCR reagents

As the PEGMA-P2VP and P2VP_32_-*b*-PBzMA_300_ particles are cationic, they have the potential to flocculate with any other negatively charged species. For example, in a PCR reaction, there are a number of reagents that are negatively charged including the primers and deoxyribonucleotide triphosphate (dNTPs). If the particles sediment in the presence of these species a ‘false positive’ result will be recorded. Hence, a series of control experiments were conducted in which the particles were added individually to different components of a PCR mastermix in order to observe whether flocculation occurred. Thus, a series of solutions containing individual reagents were prepared at the concentration they would be used at in a typical PCR. Subsequently, the PEGMA-P2VP or P2VP_32_-*b*-PBzMA_300_ latexes were added to them and observed.

For the P2VP_32_-*b*-PBzMA_300_ particles, on addition of latex to the dNTPs flocculation occurred. Sedimentation of the particles occurred within 10 minutes, as judged visually and by UV-Vis (see Fig. S5, ESI†). Therefore, this latex would result in false positives when exposing it to an unsuccessful *or* successful PCR reaction and ultimately making these particles unsuitable for this application.

**Fig. 2:**
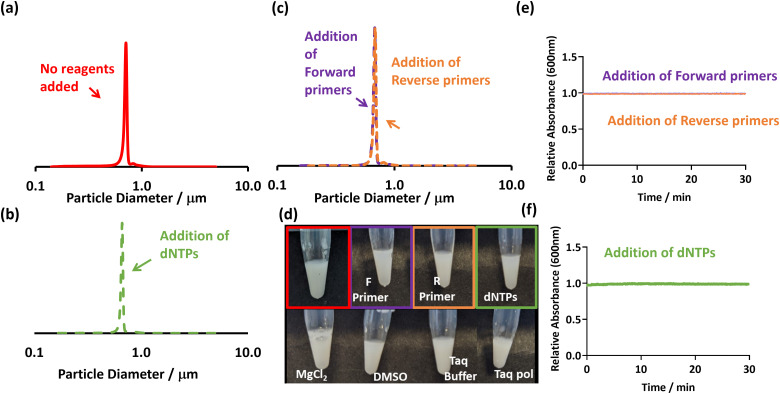
(a–c) DCP particle size distributions obtained for PEGMA-P2VP latex (0.01% w/w), showing size distributions with the absence and presence of PCR reagents. (d) Digital images of the addition of various PCR reagents to PEGMA-P2VP latex (0.2% w/w). (e–f) UV-Vis spectrophotometry showing the change in absorbance at 600 nm after the addition of individual PCR reagents to the PEGMA-P2VP latex (0.1% w/w). In all cases, the concentration of PCR reagents added were at a concentration used in a typical PCR mastermix.

For the PEGMA-P2VP particles, no indications of sedimentation occurred in the presence of dNTPs (Fig. 2b and f). In addition, the PEGMA-P2VP latex showed no signs of flocculation when any common PCR reagents were added, including primers, solvents, salts and *Taq* polymerase. In all cases, there was no change in absorption at 600 nm after being monitored for 30 minutes, the DCP traces remained mono-modal, and the dispersions remained milky white (Fig. 2).

The difference between these two latexes is likely due to their differing morphologies, with the cationic stabiliser chains losing their ability to provide stabilisation when associated with negatively charged dNTP molecules. This is supported by aqueous electrophoresis measurements which show a charge reversal from cationic to anionic when dNTPs are added (Fig. S6, ESI†). In contrast, the PEGMA-P2VP particles have a non-ionic PEGMA steric stabiliser. Therefore, although charge reversal occurs in the presence of dNTPs through their association with the P2VP core of the particles (Fig. S6, ESI†), they remain stable due to the PEGMA stabiliser. Thus, whilst the PEGMA-P2VP latexes are susceptible to bridging flocculation induced by relatively high molecular weight anionic species (in this case amplified DNA), they are not sensitive to small-molecule or salt induced aggregation. Overall, this shows that the flocculation and consequent sedimentation of the PEGMA-P2VP particles in the presence of amplified DNA (Fig. 1) is due to a combination of bridging and electrostatic flocculation.

To further investigate the role of the PEGMA stabiliser on providing stability in the presence of PCR reagents, two additional PEGMA-P2VP latexes were prepared using PEGMA with a lower average *M*_*n*_ (360 and 500 g mol^−1^, Fig. S7, ESI†). When challenged with amplified DNA these latexes also sediment but they were not stable in the presence of PCR mastermix (Fig. S8, ESI†), indicating the importance of having a suitably high molecular weight non-ionic stabiliser in avoiding false positives from occurring. Furthermore, the particle diameter of the PEGMA-P2VP latex also affected the rate and clarity of sedimentation when amplified DNA was added (Fig. S9, ESI†), with the larger particles giving the clearest visible result.

Additional negatively charged species which may be present in bacterial DNA amplification include proteins and cell/bacteria debris. Most of these species are typically removed through DNA extraction and/or purification steps. Nevertheless, when a PCR is conducted typically, the sample is lysed (heated) to release the template DNA for amplification. Thus, in order to analyse the potential interference of any bacterial cell proteins still present prior to purification, a sample of bacterial cell lysate from *P. aeruginosa* was added to the PEGMA-P2VP latex and no flocculation was observed (Fig. S10, ESI†).

### Response of PEGMA-P2VP latex to amplified DNA of varying concentration and sequence length

**Fig. 3:**
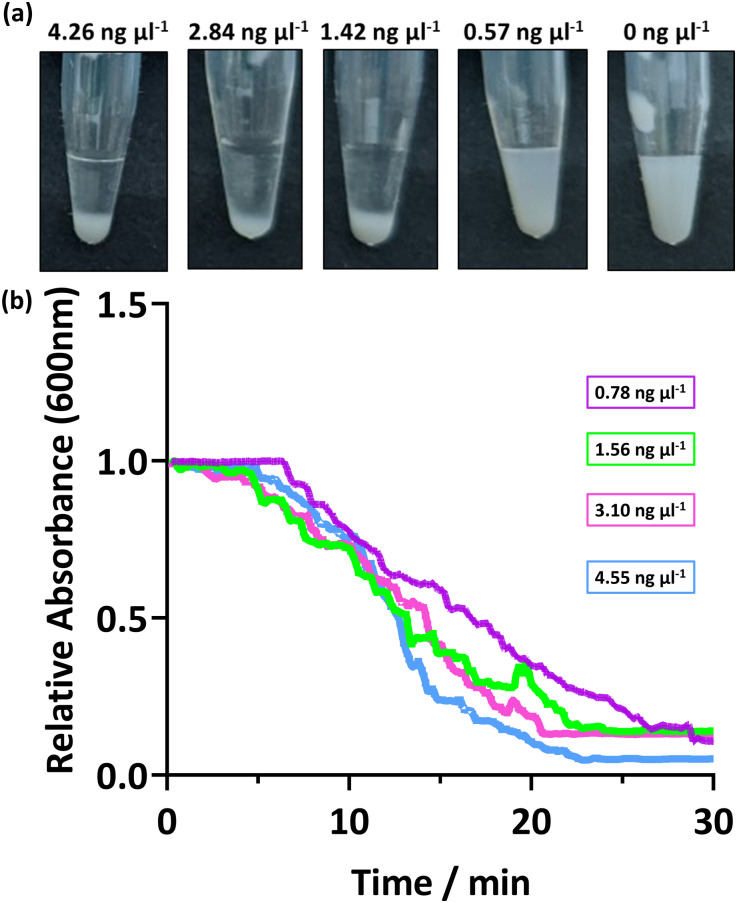
(a) Digital images showing the addition of varying concentrations of amplified DNA to PEGMA-P2VP latex (0.2% w/w). (b) UV-Vis spectrophotometry data showing the change in absorbance at 600 nm after the addition of DNA to PEGMA-P2VP latex (0.1% w/w). To vary the concentration of amplified DNA, 50 μl of purified PCR product was diluted with PCR buffer.

The response of the PEGMA-P2VP latexes for the detection of amplified DNA was investigated using different concentrations and amplified DNA sequence lengths. This is important as when undertaking a PCR reaction, the quantity of amplified DNA at the end of the cycle can vary, and the number of cycles directly affects the obtained DNA concentration. Thus, the bacterial 16s ribosome from *P. aeruginosa* was amplified by colony PCR using universal primers (27F, 1492R) and the concentration determined. This amplified DNA was subsequently diluted to provide a range of concentrations before addition to the PEGMA-P2VP latex (0.1 and 0.2% w/w latex for UV-Vis and visual analysis, respectively). As shown in Fig. 3a, visible sedimentation is apparent at amplified DNA concentrations between 4.26 to 1.42 ng μl^−1^. At 0.57 ng μl^−1^, visual inspection indicates that some sedimentation occurred but the dispersion is still relatively milky. In a real-world setting, this would potentially be construed as either a negative or positive result and as such forms a sensitivity limit for this combination of latex and amplified DNA. The DNA yield obtained in the PCR reaction depends on a number of factors, such as the size of the amplicon, starting amount of template and amplification parameters. For example, a typical PCR reaction produces a final amplified DNA concentration between 15 to 25 ng μl^−1^, but can be as low as 3 ng μl^−1^.^24^

Fig. 3b shows the sedimentation rate of the PEGMA-P2VP latexes for different amplified DNA concentrations. In all cases, the measured absorbance of the 0.1% latex dispersion decreases steadily over 20–30 minutes, with all samples analysed reaching ∼0 absorbance after 30 minutes. Thus, for this PEGMA-P2VP latex and amplified DNA combination, 30 minutes is sufficient to judge the outcome of a test by allowing the particles to fully sediment to the bottom of the tube. However, when observing flocculation by eye, the presence of flocculation is usually apparent within a few minutes.

**Fig. 4:**
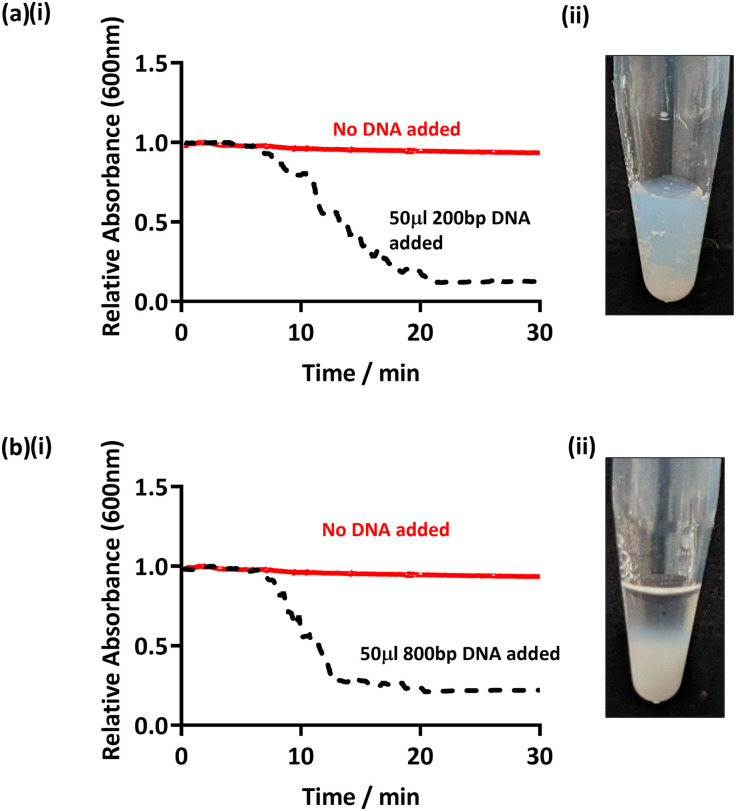
(a) UV-Vis spectrophotometry (i) and digital image (ii) after the addition of amplified DNA with a sequence length of 200 bp to PEGMA-P2VP latex. (b) UV-Vis spectrophotometry (i) and digital image (ii) after the addition of amplified DNA with a sequence length of 800 bp. Latex concentrations were 0.1% w/w and 0.2% w/w for UV-Vis and digital image studies, respectively, and an overall DNA concentration of 3 ng μl^−1^ was used in all cases.

In order to assess whether this method can be applied to amplified DNA with shorter sequence lengths, PCR targeting other regions of the bacterial genome was conducted and the products added to PEGMA-P2VP latex. Specifically, partial 16S rRNA gene sequences were used to amplify DNA of an approximate base pair length of 200 and 800 bp (see Table 2).^38,39^ Hence, purified PCR products were added to the PEGMA-P2VP particles (latex concentration 0.2% w/w, DNA concentration 2–3 ng μl^−1^) and observed for 30 minutes (Fig. 4). In both cases, flocculation was observed within 20 min visibly and by UV-Vis monitoring. DCP analysis (Fig. S11, ESI†) also showed the presence of a shoulder due to flocculation on the addition of amplified DNA of both sequence lengths. However, the clarity of the supernatant observed for 200 bp (Fig. 4a ii) was not as clear as previously observed for longer amplified DNA sequence lengths. This is likely due to the shorter amplified DNA length being a less efficient flocculant. This indicates that this particular latex/DNA combination (concentration *etc.*) requires further optimisation for detecting relatively short amplified DNA sequence lengths.

### Response of PEGMA-P2VP latex to fungal DNA control

**Fig. 5:**
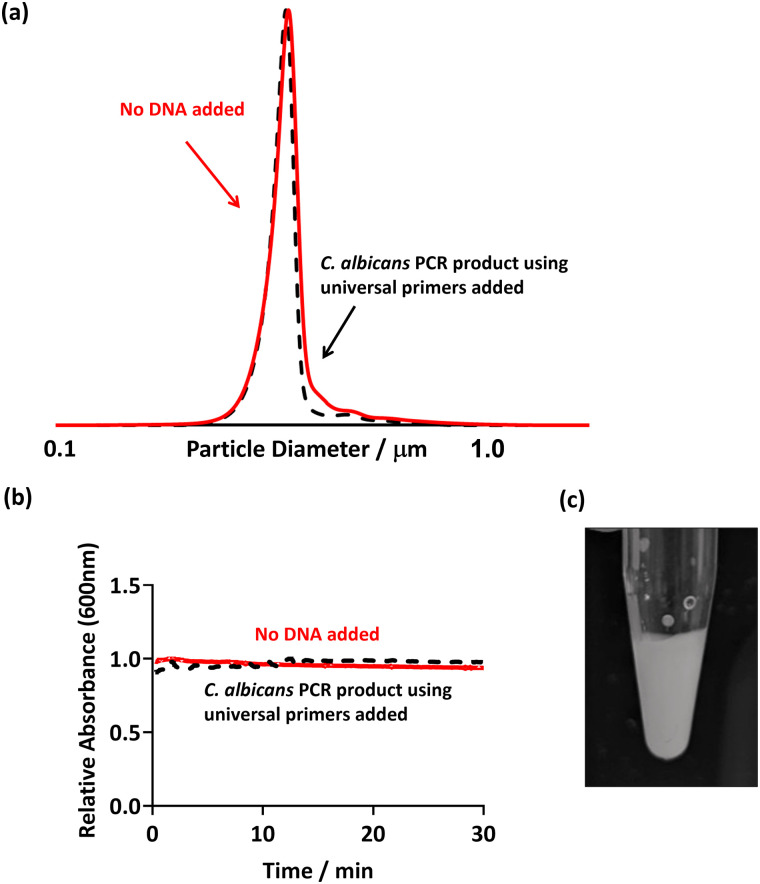
(a) DCP particle size distribution obtained for PEGMA-P2VP latex (0.01% w/w) showing size distribution with absence and presence of fungal *C. albicans* PCR product. (b) UV-Vis spectrophotometry showing the change in absorbance at 600 nm after the addition of *C. albicans* PCR product to the PEGMA-P2VP latex (0.1% w/w). (c) Digital image of the addition of 50 μl PCR product using *C. albicans* as template DNA and PEGMA-P2VP latex (0.2% w/w). In all cases, universal bacterial primers were used in the PCR mastermix.

In order to show that false positives would not be recorded in the presence of non-targeted pathogens, PCR was conducted using universal bacterial primers in the presence of a sample from a fungal species. *Candida albicans* was used as the template, and the PCR was run as previously discussed (using 27F 1492R universal primers, Table 2). Thus, in theory, no DNA should be amplified as the primers used are specific to bacteria and not to eukaryotic fungal species such as *C. albicans*. This PCR product was then purified and PEGMA-P2VP latex was added as before (Fig. 5).

Flocculation of the latex particles did not occur as evidenced by no changes in: (i) the DCP trace of the PEGMA-P2VP latex after the addition of PCR product (Fig. 5a); (ii) the UV-Vis absorption over the course of 30 minutes (Fig. 5b); and visual inspection of the PCR tube (Fig. 5c). Hence, this confirms that flocculation only occurs when amplified DNA is present as a result of a successful PCR.

### Prospects

Herein it has been demonstrated that this methodology could provide a user friendly, point-of-care initial test for bacterial infections. A range of potential applications include eye scrapes, wounds and blood cultures, which would assist the immediate treatment of infections. For example, in optometry clinics this may be done at the point-of-care to determine whether an eye infection is bacterial as opposed to being viral, fungal, or amoebal in nature. Hence the correct course of treatment can be prescribed, infections can be resolved quickly, and the unneeded use of antibiotics can be reduced. Furthermore, by using a portable PCR thermocycler, it would be possible to apply this methodology to relatively remote, difficult to access, or time critical areas such as military camps or in developing countries, as this is where more advanced facilities and expertise are not available.

Since the detection of amplified DNA by this method relies on a successful PCR being carried out, there is the potential to utilise this simple detection method for rapid speciation or detection of specific genetic motifs, by changing the primer set used. For example, PCR can be used to amplify DNA from viruses and fungi, as well as targeting *e.g.* MRSA specific genes in bacteria to best identify a course of treatment, which is being pursued currently.

## Conclusions

The detection of the presence of amplified DNA from bacterial samples using a sterically-stabilised, cationic polymer latex and widely available equipment is demonstrated, providing an accessible alternative DNA detection technique. If DNA is present in a sample, successful amplification by polymerase chain-reaction (PCR) results in the amplified DNA inducing flocculation of the polymer latex followed by rapid sedimentation. Specifically, four latex morphologies were prepared (anionic, non-ionic and two cationic) and individually added to amplified DNA from *P. aeruginosa*. Both the cationic latexes (PEGMA-P2VP, P2VP_32_-*b*-PBzMA_300_) were shown to flocculate in the presence of amplified PCR product using digital images, DCP and UV-Vis spectrophotometry, whereas there was no change observed with the anionic and non-ionic latexes. In order to analyse whether these particles could be used to detect amplified DNA, individual PCR reagents were added to the latexes and flocculation was analysed. The P2VP_32_-*b*-PBzMA_300_ particles were shown to flocculate with the negatively charged dNTPs that were present in the PCR mastermix. PEGMA-P2VP particles with a suitably high molecular weight stabiliser did not flocculate in the presence of any PCR reagents and therefore could be used for further experiments to detect amplified DNA. This PEGMA-P2VP latex was shown to flocculate with varying concentrations of DNA, showing a visible sedimentation of DNA down to 0.78 ng μl^−1^, with amplified DNA sequence lengths of 200 and 800 bp. As a control experiment, fungal DNA from *C. albicans* was used as the template DNA in the PCR reaction, with the primers targeted to the 16s ribosomal gene for bacteria. DNA was not amplified as these primers are specific to bacteria and therefore no flocculation was observed when PEGMA-P2VP latex was added to this PCR product, due to the absence of DNA, which would cause flocculation. Overall, this flocculation mechanism is thought to be due to bridging and electrostatic flocculation and could be used to detect amplified DNA in a wide range of settings.

## Experimental

### Synthesis of PEGMA-stabilised P2VP latex

The preparation of poly(ethylene glycol) methyl ether methacrylate (PEGMA)-stabilised P2VP latexes *via* aqueous emulsion polymerisation has been reported in detail previously.^40–42^ Aliquat 336 (0.5 g; Thermo-Fisher, UK) and PEGMA stabiliser (1.0 g, average *M*_*n*_ 2000 g mol^−1^; Sigma-Aldrich, UK) were added to a 100 ml single necked round bottomed flask and dissolved in 38.5 g of deionised water by stirring at 250 rpm with a magnetic stirrer. 2-Vinylpyridine (2VP, 4.95 g; Sigma-Aldrich, UK) and divinylbenzene (DVB, 0.05 g; Sigma-Aldrich, UK) were added *via* syringe as a comonomer mixture. The round bottomed flask was then sealed, and the solution was degassed using five vacuum/nitrogen cycles using a Schlenk line. This was continually stirred at 250 rpm using a magnetic stirrer and then heated to 60 °C in an oil bath. 0.085 g of 2,2′-azodiisobutyramidine dihydrochloride (AIBA; Sigma-Aldrich, UK) was dissolved in 5 g of deionised degassed water and added to the reaction vessel after 20 minutes of stirring and heating. The polymerisation was allowed to proceed for 12 h at 60 °C. In subsequent reactions the particle diameter was varied by changing the AIBA concentration and the molecular weight of the stabiliser was varied using PEGMA with an average *M*_*n*_ of 360 and 500 g mol^−1^. The obtained latexes were purified by placing dispersions into a sealed dialysis membrane (Spectrum™ Spectra/Por™ 3 RC Dialysis Membrane Tubing 3500 Dalton MWCO, Fisher Scientific, UK), and immersing them in 1000 ml of deionised water. The deionised water was changed twice daily until the surface tension of the water was 71 ± 1 mN m^−1^, as judged by pendant drop analysis.

### Dynamic light scattering (DLS)

DLS studies were conducted at 25 °C using a Malvern Zetasizer. Back-scattered light was detected at 173° and the mean hydrodynamic particle diameter was calculated over twenty runs of ten seconds duration from the quadratic fitting of the correlation function using the Stokes–Einstein equation. All measurements were performed in triplicate at 0.01% w/w in deionised water.

### Disc centrifuge photosedimentometry (DCP)

DCP analyses were conducted using a Centrifugal Photo Sedimentation (CPS) Disc Centrifuge Model 24 000. A density gradient that ranged from 12 to 4% w/w sucrose solution in deionised water was constructed and allowed to stabilise for approximately 30 minutes. A 345 nm polystyrene latex calibration standard was injected prior to the analysis of each sample. Run times were between 5 to 30 minutes with the centrifugation rate typically 20 000–23 000 rpm.

### Aqueous electrophoresis

Zeta potential measurements were conducted in the presence of 1 mM KCl using a Malvern Zetasizer. The zeta potential was calculated from the electrophoretic mobility using the Smoluchowski relationship. The solution pH was adjusted by adding either dilute (0.01–0.1 M) HCl or KOH. All measurements were taken on 0.01% w/w dispersions that had been equilibrated at the desired pH for at least 20–40 min.

### UV-visible spectrophotometry

UV-Vis absorption spectra were recorded on Agilent Cary 60 UV-Vis spectrophotometer at 600 nm at room temperature. The concentration and purity of the DNA was assessed using a Nanodrop 2000 spectrophotometer (Thermo Scientific Nanodrop ND2000 s/n Q372) or with the use of a Agilent Cary 60 UV-Vis spectrophotometer. Data was analysed and a moving average was calculated and normalised using the ‘Normalise’ function on GraphPad Prism 9.

### Transmission electron microscopy (TEM)

TEM studies were conducted using a FEI Tecnai G2 20 instrument operating at 200 kV and equipped with a Gatan 1 k CCD camera. Aqueous polymer dispersions were diluted to approximately 0.1% w/w using deionised water at ambient temperature. TEM samples were prepared by depositing 2 μL of diluted copolymer dispersion onto carbon-coated copper grids (Agar Scientific, 400 mesh) and dried under ambient conditions for 30 min.

### Polymerase chain reaction (PCR)

Experiments were conducted using either a reference strain of *Pseudomonas aeruginosa* PA01^43,44^ or *Candida albicans* ATCC 10 231. These strains were incubated overnight at 37 °C on nutrient agar plates. DNA was extracted using QIAamp DNA mini kit (Qiagen, UK), using one bacterial or fungal colony. Alternatively, bacterial colonies were transferred to 20 μl of sterile MilliQ water in a PCR tube and boiled for 10 minutes, with the resultant lysate used as the template for the subsequent PCR reaction, as described previously.^45^

For a typical PCR, per sample the following reagents were added to a 0.2 ml PCR tube; 35.25 μl of molecular water (Sigma-Aldrich, UK), 5 μl of PCR buffer (Taq Buffer (10X), without detergent; Thermo-Fisher, UK), 1.5 μl MgCl_2_ (50 mM; Meridan Bioscience, UK), 1 μl dNTPs (10 mM; Thermo-Fisher, UK), 2.5 μl forward primer (Eurofins Genomics, EU), 2.5 μl reverse primer (Eurofins Genomics, EU), 1 μl DMSO (Sigma-Aldrich, UK), 1 μl DNA template and 0.25 μl *Taq* polymerase (Invitrogen; Thermo-Fisher, UK). PCR was conducted using a TGradient PCR instrument (Biometra Göttingen, Germany). PCR tubes were placed into the pre-programmed thermal cycler to start the PCR reaction. The 16S programmed cycle was set as follows; stage 1 – 95 °C for 2 min; stage 2 – 95 °C for 1 min, 53 °C for 30 s, 72 °C for 1 min (repeated for 30 cycles); stage 3 – 72 °C for 5 min. Following the PCR cycle, 10 μl of PCR product was mixed with 2 μl loading dye (Thermo-Fisher, UK) and analysed by gel electrophoresis on a 1% w/v agarose gel at 120 V for 90 minutes. If required, purification of the PCR product was performed using QIAquick PCR purification Kit (Qiagen, UK). The DNA gene targeted was the 16S rDNA gene using the following primers: forward primer 27F (5′-AGA GTT TGA TCC TGG CTC AG-3′); reverse primer 1492R (5′-TAC CTT GTT ACG ACT T-3′). Therefore, the amplified PCR product was approximately 1400 base pairs in length. In subsequent experiments, the DNA sequence length was varied using the following primers: 8FPL1 (5′-GAG TTT GAT CCT GGC TCA G-3′) and 806R (5′-GGA CTA CCA GGG TAT CTA AT-3′) to amplify DNA of an approximate base pair length of 800bp; HDA1 (5′-ACT CCT ACG GGA GGC AGC AGT-3′) and HDA2 (5′-GTA TTA CCG CGG CTG CTG GCA C-3′) which target the V2-V3 region of the 16s rRNA gene to give an overall base pair sequence of approximately 200bp.

### Detection of amplified bacterial DNA

Latex particles were added to solutions of purified PCR product in their PCR tubes to give an overall latex concentration of 0.2% w/w. The mixture of latex and DNA was then left undisturbed at room temperature for 30 minutes, before digital photographs were taken. For the UV-Vis experiments, latexes were diluted to 0.1% w/w in a cuvette, and on addition of DNA data was collected over a 30 minute period. For DCP experiments, latex was diluted to 0.01% w/w and added to the DNA sample. This was then left for 30 minutes, before analysis of the data.

## Conflicts of interest

There are no conflicts to declare.

## Supplementary Material

TB-011-D2TB02714C-s001
