# Supplementary material for: Visible label-free detection of bacterial DNA using flocculation of sterically stabilised cationic latexes
Source: J Mater Chem B. 2023 Mar 14;11(17):3787–96. doi: 10.1039/d2tb02714c (PMC10155078; doi:10.1039/d2tb02714c)
Supplement: TB-011-D2TB02714C-s001 [file TB-011-D2TB02714C-s001.pdf]

Electronic Supplementary Material (ESI) for Journal of Materials Chemistry B.
This journal is © The Royal Society of Chemistry 2023

Electronic Supporting Information for:

Visible label-free detection of bacterial DNA using flocculation of sterically stabilised cationic latexes

*Elisabeth Trinh [a], Kate L. Thompson [a], Shang-Pin Wen [a], Gavin J. Humphreys [b], Bianca L. Price [b, c], Lee A. Fielding [a, d, *]*

a. Department of Materials, School of Natural Sciences, The University of Manchester, Oxford Road, Manchester, M13 9PL, UK.
b. Division of Pharmacy and Optometry, School of Health Sciences, Faculty of Biology Medicine and Health, The University of Manchester, Oxford Road, Manchester, M13 9PT, UK.
c. Division of Pharmacy and Optometry, Lydia Becker Institution of Immunology and Inflammation, Faculty of Biology Medicine and Health, The University of Manchester, Oxford Road, Manchester, M13 9PT, UK.
d. Henry Royce Institute, The University of Manchester, Oxford Road, Manchester, M13 9PL, UK.
** lee.fielding@manchester.ac.uk*

Materials and additional experimental methods

Materials

2-Vinylpyridine (97%, 2VP; Sigma-Aldrich, UK), divinylbenzene (80 mol % 1,4-divinyl content, DVB; Sigma-Aldrich, UK) and benzyl methacrylate (BzMA, 98%, Sigma-Aldrich, UK) were passed through a column of activated basic alumina to remove inhibitors and impurities before use. Hexadecane (HD, 99%) was purchased from Alfa Aesar (UK). Diethyl ether (99%), azobisisobutyronitrile (AIBN, 98%), and 2,2'-azodiisobutyramidine dihydrochloride (AIBA; 97%) were purchased from Sigma-Aldrich (UK) and used as received. Aliquat 336 surfactant was purchased from Fisher Scientific (UK) and used as received. Lutensol TO 20, which is a saturated iso-C13 alcohol with an ethoxylation block length of 20 units was obtained from BASF (Germany) and used as received. Poly(ethylene glycol) methyl ether methacrylate (PEGMA, average M_n 2,000 g mol^{-1}, Sigma-Aldrich, UK) was supplied as a 50 wt. % solution in H_2O. PEGMA with an average M_n 360 g mol^{-1} and average M_n 500 g mol^{-1} (Sigma Aldrich, UK) were passed through a basic alumina column to remove inhibitors before use. Bis-(2-phenylethanesulfanyl thiocarbonyl) disulphide (PETTCCP) was prepared in-house using previously published methods.[1] Deionised water was used throughout this study for analysis and dilution. PCR reagents were used as received. DNA extraction and purification kits (Qiagen, UK) were used as per manufacturer's instructions.

Preparation of poly(benzyl methacrylate) by RAFT miniemulsion polymerisation

The synthesis of poly(benzyl methacrylate) with a mean degree of polymerisation of 200 (DP) *via* reversible addition–fragmentation chain transfer (RAFT) miniemulsion polymerisation was conducted at a dispersed phase content of 20 % w/w, as previously reported.[2] BzMA (7.8 g, 44.087 mmol); PETTCCP (41.4 mg, 0.147 mmol); AIBN (4.8 mg, 0.029 mmol, [Chain Transfer Agent (CTA)]/[initiator] = 5); Lutensol TO 20 (604.0 mg, 7.8% w/w related to BzMA); HD (185.2 mg, 2.4% w/w related to BzMA); and water (31.4 g) were used. The reactor contents were deoxygenated by purging with nitrogen for 10 min at room temperature. After deoxygenation, the round-bottomed flask was immersed into a preheated oil bath at 70 °C for 240 min. Polymerisations were quenched by cooling to room temperature and exposing to air.

Preparation of poly(4-styrene sulfonate)-block-poly(benzyl methacrylate) (PSS-*b*-PBzMA) diblock copolymer nanoparticles *via* RAFT mediated polymerisation-induced self-assembly (PISA)

Previous work on the synthesis of these diblock copolymer nanoparticles has been described previously.[1] PSS_{54}-*b*-$PBzMA_{100}$ latexes were synthesised at 70°C for 24hr using PSS_{54} macro-CTA (85.2 mg, 0.012 mmol), BzMA (215.3mg, 1.22 mmol), ACVA (1.1 mg, 0.004 mmol, CTA/initiator molar ratio = 3) in an 80 % methanol/water mixture. Polymerisations were quenched by cooling to room temperature and exposing to air.

Synthesis of poly(2-vinyl pyridine)-block-poly(benzyl methacrylate) P2VP–*b*-PBzMA diblock copolymer nanoparticles *via* RAFT emulsion polymerisation

Previous work on the synthesis of these diblock copolymer nanoparticles has been described previously.[3] $P2VP_{32}$–$PBzMA_{300}$ was synthesised in water at pH 2, using $P2VP_{32}$ macro-CTA (32.3 mg, 0.009 mmol), AIBA (0.8 mg, 0.003 mmol, CTA/initiator molar ratio = 3), and deionised water. The solution pH was slowly adjusted to pH 2 using an aqueous solution of 0.1 M HCl, and then BzMA (467.7 mg, 2.654 mmol) was added. The vial was sealed and deoxygenated by purging nitrogen for 10 min before being placed in a preheated oil bath at 70 °C for 24 h to ensure complete conversion of BzMA. Polymerisations were quenched by cooling to room temperature and exposing to air.

Supporting Figures

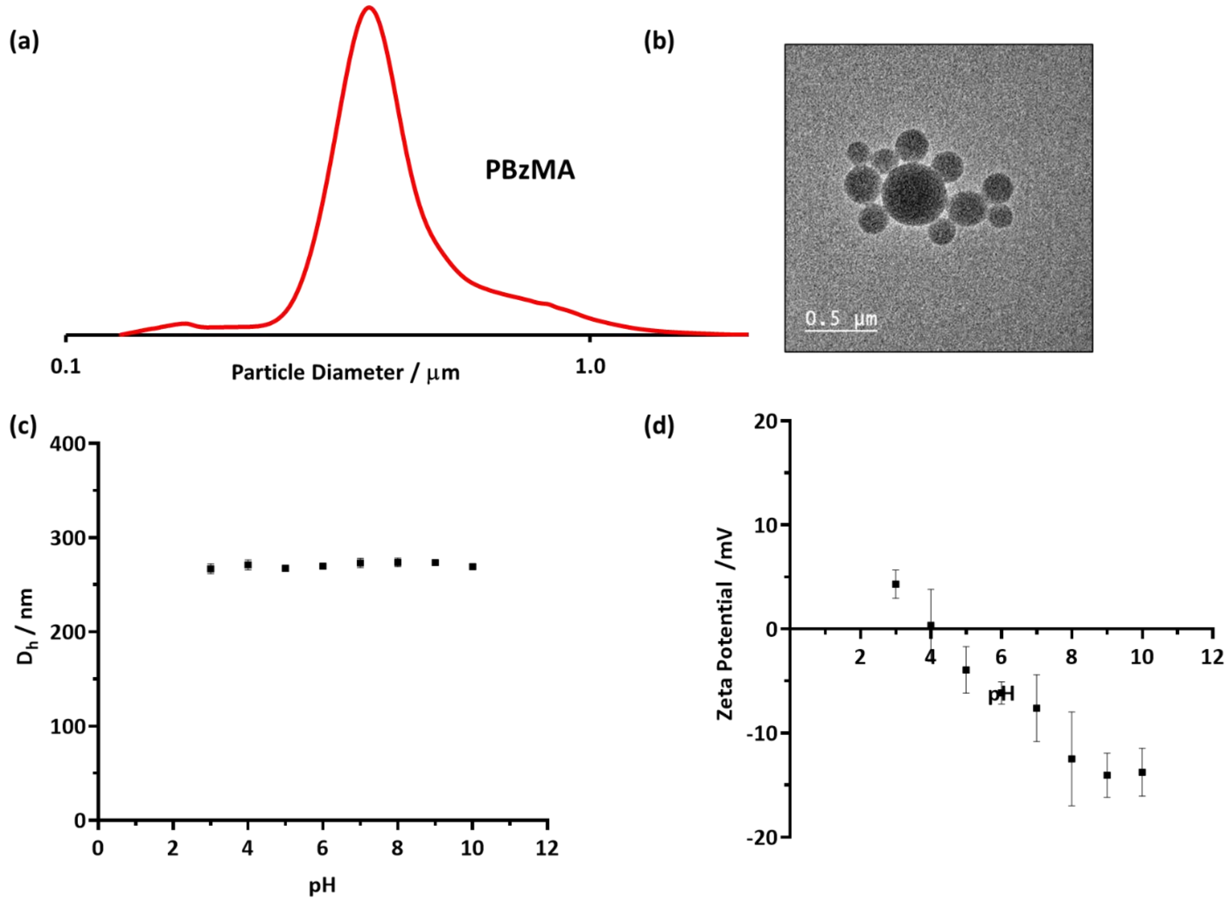

Figure S1. Characterisation of non-ionic PBzMA latex particles prepared by miniemulsion polymerisation. (a) Disc Centrifuge Photosedimentometry (DCP) particle size distributions were calculated using a particle density of 1.18 g cm^{-3}. (b) Representative TEM image. (c) Mean hydrodynamic diameter and (d) zeta potential as a function of pH. Measurements were conducted at a latex concentration of approximately 0.01 % w/w with 1 mM KCl as a background electrolyte. The pH was adjusted using KOH and HCl.

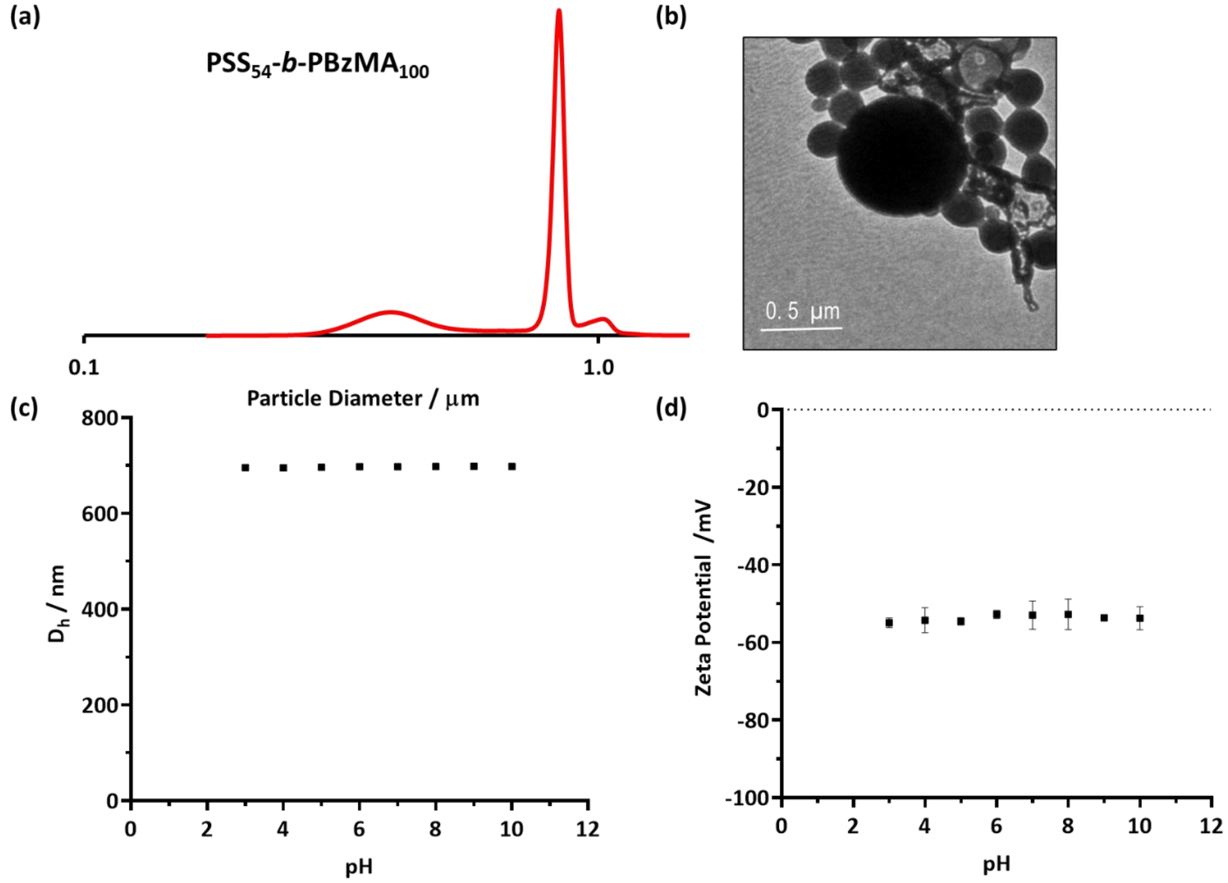

Figure S2. Characterisation of anionic PSS$_{54}$-*b*-PBzMA$_{100}$ latex particles prepared by RAFT mediated PISA. (a) DCP particle size distributions were calculated using a particle density of 1.18 g cm^{-3}. (b) Representative TEM image. (c) Mean hydrodynamic diameter and (d) zeta potential as a function of pH. Measurements were conducted at a latex concentration of approximately 0.01 % w/w with 1 mM KCl as a background electrolyte. The pH was adjusted using KOH and HCl.

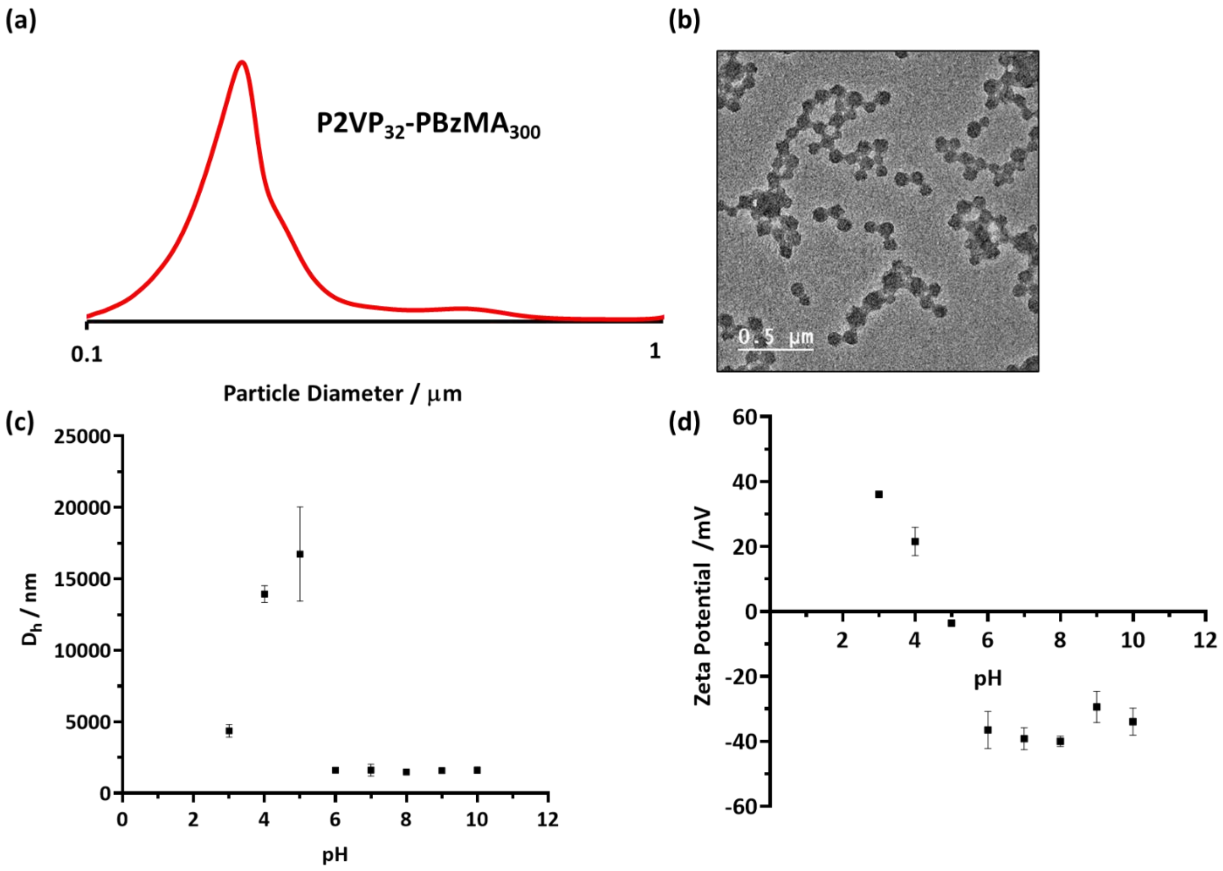

Figure S3. Characterisation of $P2VP_{32}$-*b*-$PBzMA_{300}$ latexes prepared by RAFT emulsion polymerisation. (a) DCP particle size distributions were calculated using a particle density of 1.18 g cm^{-3}. (b) Representative TEM image. (c) Mean hydrodynamic diameter and (d) zeta potential as a function of pH. Measurements were conducted at a latex concentration of approximately 0.01 % w/w with 1 mM KCl as a background electrolyte. The pH was adjusted using KOH and HCl.

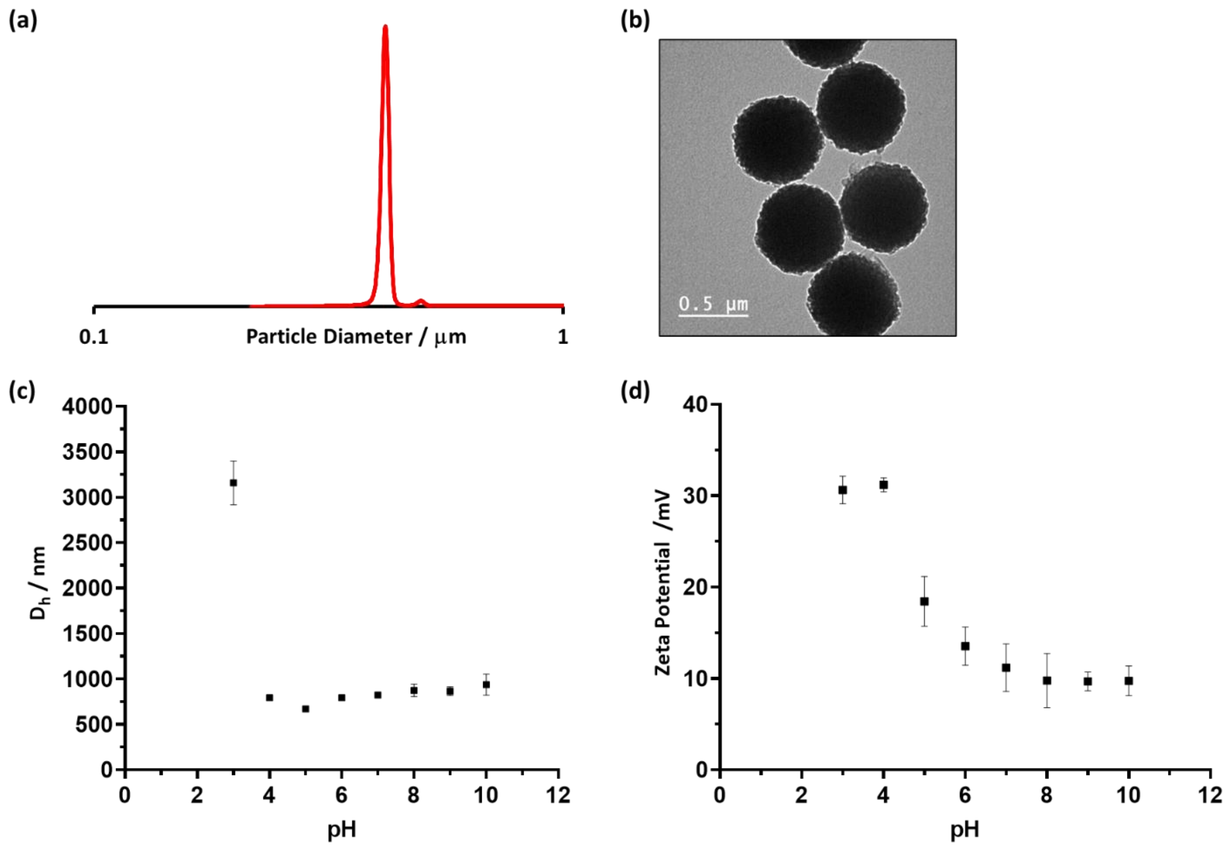

Figure S4. Characterisation of ~700 nm cationic sterically-stabilised PEGMA-P2VP latex particles prepared by emulsion polymerisation. (a) DCP particle size distributions were calculated using a particle density of 1.18 g cm^{-3}. (b) Representative TEM image. (c) Mean hydrodynamic diameter and (d) zeta potential as a function of pH. Measurements were conducted at a latex concentration of approximately 0.01 % w/w with 1 mM KCl as a background electrolyte. The pH was adjusted using KOH and HCl.

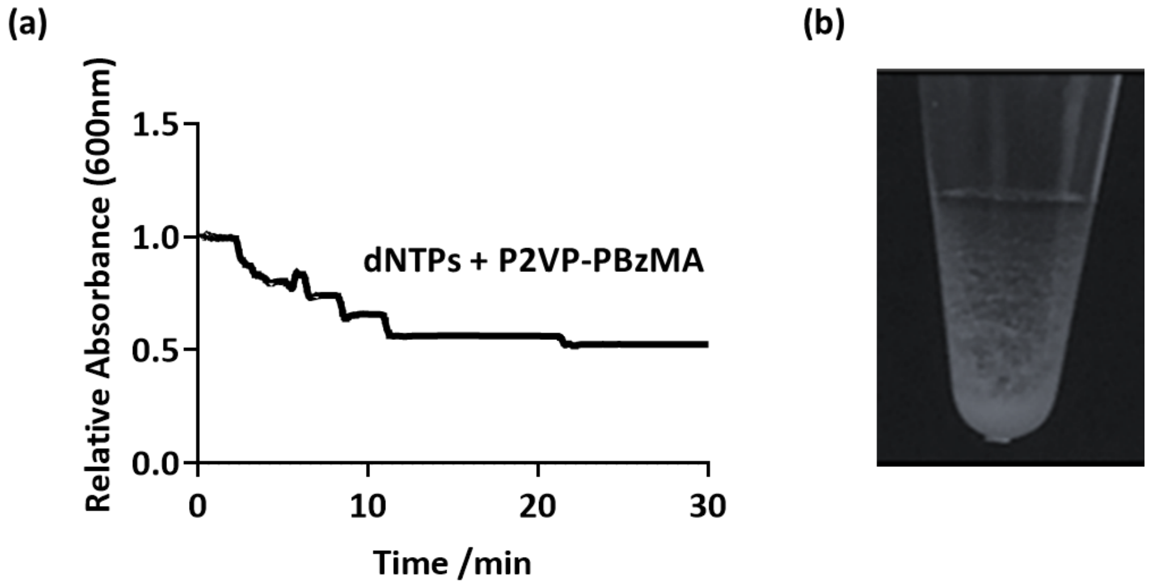

Figure S5. (a) UV-VIS spectrophotometry measurement showing the change in absorbance at 600 nm after the addition of dNTPs to a $P2VP_{32}$-*b*-$PBzMA_{300}$ latex. Latex particles were at a concentration of 0.1 % w/w and dNTPs were added at the concentration used in the PCR reaction. (b) Digital image showing flocculation after the addition of dNTPs to this latex.

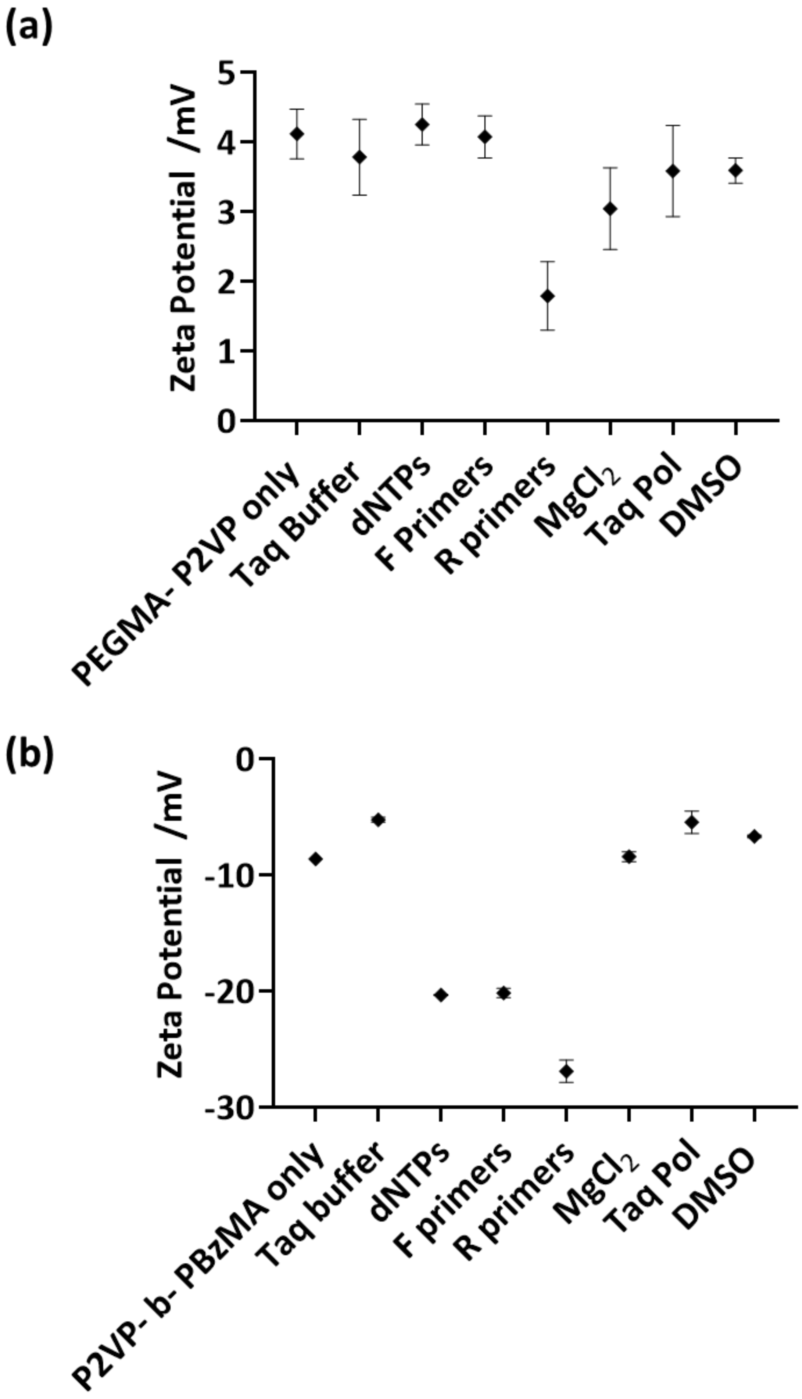

Figure S6. Zeta potential measured after the addition of individual PCR reagents to (a) sterically-stabilised PEGMA-P2VP (~700 nm) and (b) $P2VP_{32}$-*b*-$PBzMA_{300}$ latexes. Measurements were conducted at a latex concentration of approximately 0.01 % w/w with 1 mM KCl as a background electrolyte. PCR reagents were added at the concentration used in typical PCR mastermix.

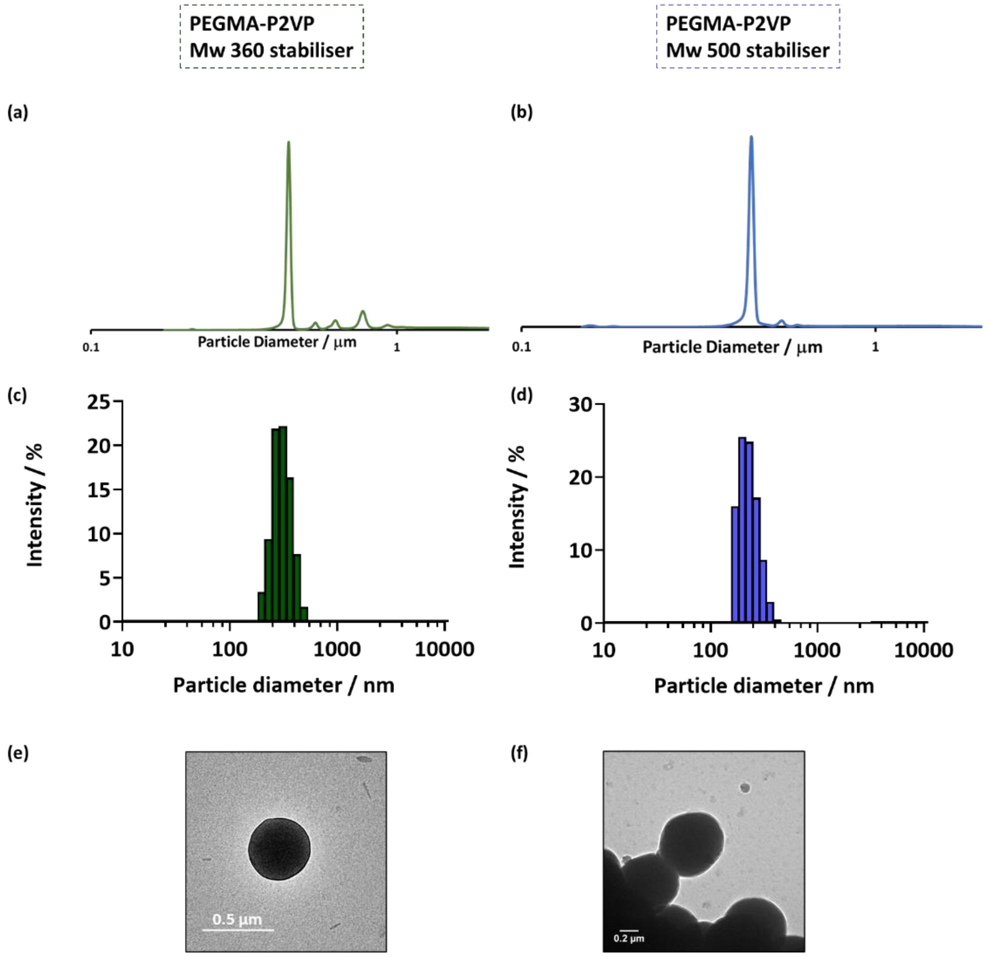

Figure S7. Characterisation of cationic sterically-stabilised PEGMA-P2VP latex particles prepared by emulsion polymerisation using PEGMA with an average M_n 360 g mol^{-1} or 500 g mol^{-1}. (a-b) DCP particle size distributions were calculated using a particle density of 1.18 g cm^{-3}. (c-d) Mean hydrodynamic diameter determined using dynamic light scattering. Measurements were conducted at a latex concentration of approximately 0.01 % w/w. (e-f) Representative TEM images.

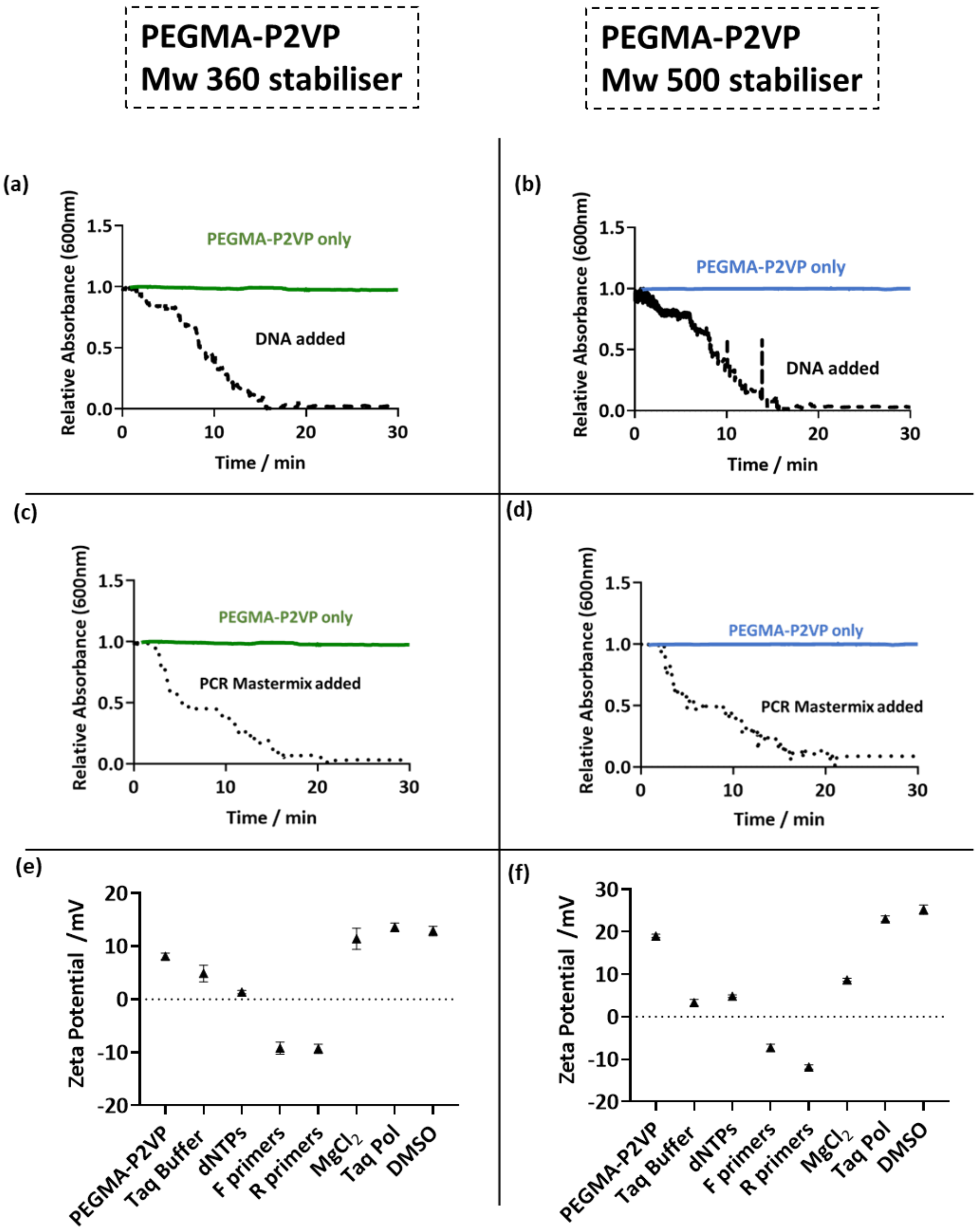

Figure S8. Behaviour of cationic sterically-stabilised PEGMA-P2VP latex particles prepared by emulsion polymerisation using PEGMA with an average M_n 360 g mol^{-1} or 500 g mol^{-1}. (a and b) UV-VIS spectrophotometry measurements showing the change in absorbance at 600 nm after the addition of amplified DNA (1400 bp). Latex particles were at a concentration of 0.1 % w/w and DNA was added at a concentration of ~4 ng µl^{-1}. (c and d) UV-VIS spectrophotometry measurements showing the change in absorbance at 600 nm after the addition of 50 µl PCR mastermix. Latex particles were at a concentration of 0.1 % w/w and PCR mastermix was added at the concentration used in a typical PCR. (e and f) Zeta potential measured after the addition of individual PCR reagents. Latex concentration of approximately 0.1 % w/w and PCR reagents were added at the concentration used in typical PCR mastermix.

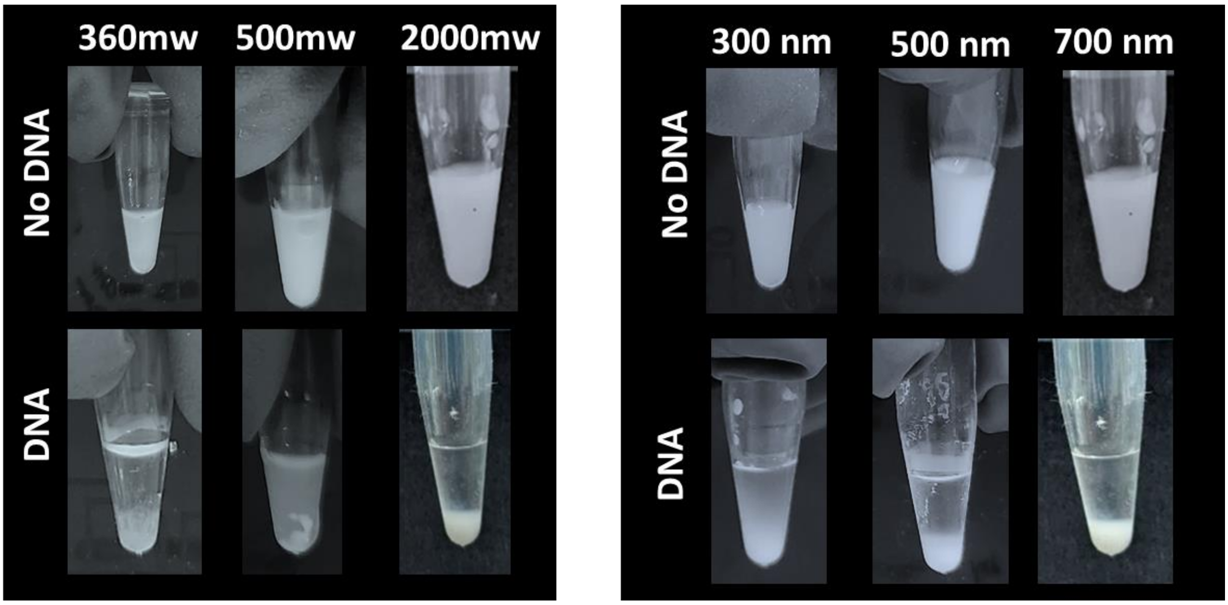

Figure S9. Digital images showing flocculation of PEGMA-P2VP latexes 30 minutes after the addition of amplified DNA from *P. aeruginosa* (1400 bp, ~4 ng µl $^{-1}$). Left: PEGMA-P2VP latexes prepared by using PEGMA with an average M_n 360, 500 and 2000 g mol^{-1}. Right: PEGMA-P2VP latexes with mean diameters between approximately 300 to 700 nm, prepared using PEGMA with M_n 2000 g mol^{-1}.

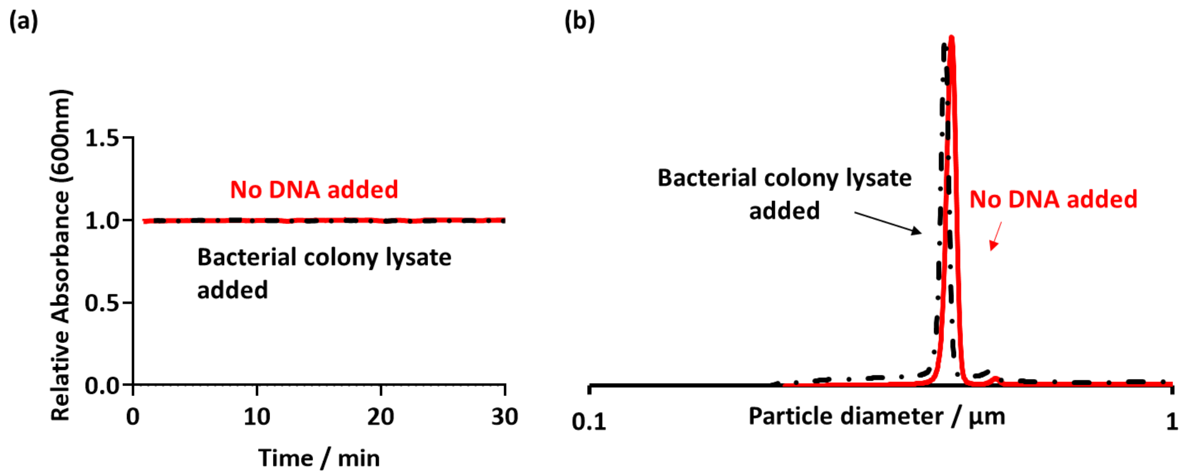

Figure S10. (a) UV-VIS spectrophotometry measurement showing the change in absorbance at 600 nm after the addition of bacterial colony lysate to PEGMA-P2VP latex (~700 nm diameter). (b) DCP particle size distributions for PEGMA-P2VP latex with the addition of bacterial colony lysate from *P. aeruginosa.*

(a)

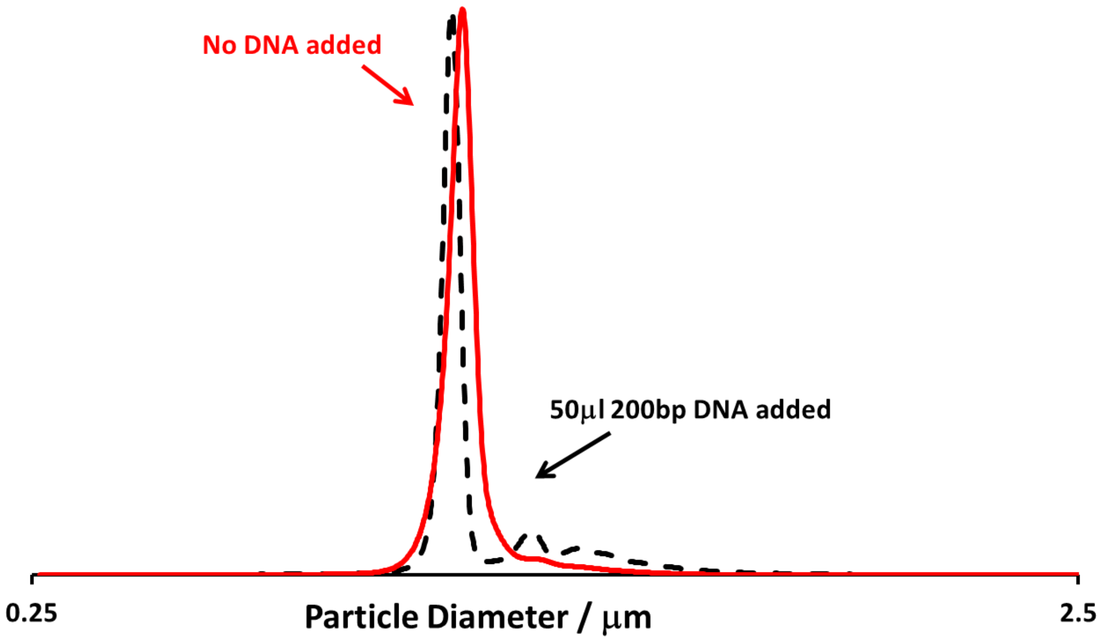

(b)

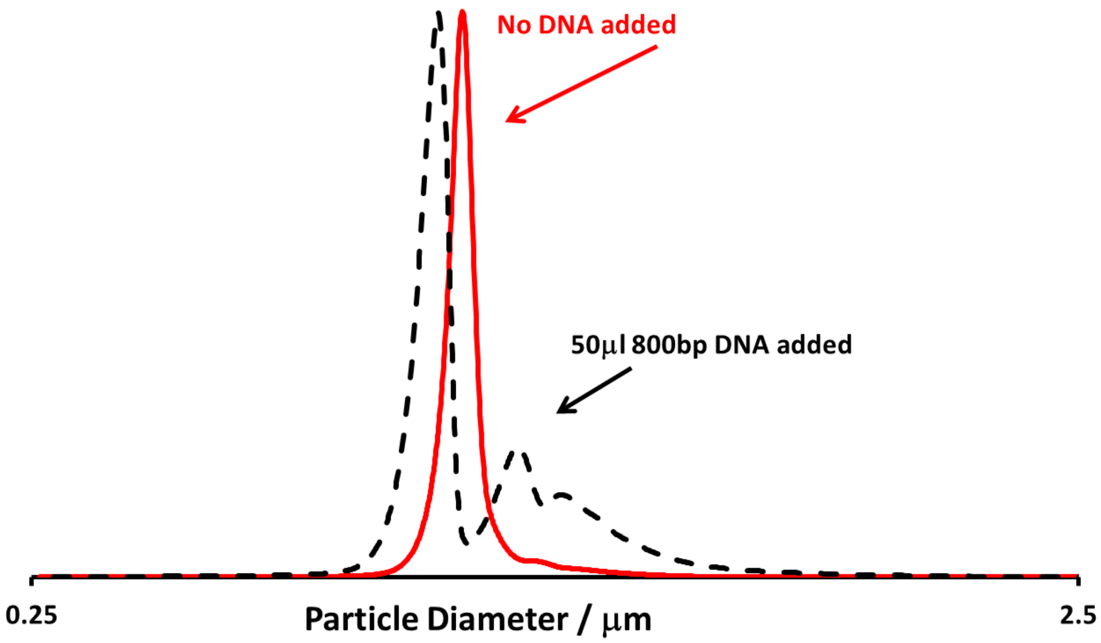

Figure S11. DCP particle size distributions for PEGMA-P2VP latex with the addition of amplified DNA from *P. aeruginosa* with (a) 200 bp and (b) 800 bp sequence lengths. Latex concentrations were 0.01 % w/w and an overall DNA concentration of 3 ng μl^{-1} was used in both cases.

References

1 S. P. Wen, J. G. Saunders and L. A. Fielding, *Polym. Chem.*, 2020, **11**, 3416–3426.

2 S. P. Wen, Q. Yue and L. A. Fielding, *Polym. Chem.*, 2021, **12**, 2122–2131.

3 S. P. Wen and L. A. Fielding, *Soft Matter*, 2022, **18**, 1385–1394.
